# SUMOylation does not affect cardiac troponin I stability but alters indirectly the development of force in response to Ca^2+^

**DOI:** 10.1111/febs.16537

**Published:** 2022-06-08

**Authors:** Bracy Fertig, Jiayue Ling, Edgar E. Nollet, Sara Dobi, Tara Busiau, Kiyotake Ishikawa, Kelly Yamada, Ahyoung Lee, Changwon Kho, Lauren Wills, Amy J. Tibbo, Mark Scott, Kirsten Grant, Kenneth S. Campbell, Emma J. Birks, Niall MacQuaide, Roger Hajjar, Godfrey L. Smith, Jolanda van der Velden, George S. Baillie

**Affiliations:** 1 Institute of Cardiovascular and Medical Sciences, College of Veterinary, Medical and Life Sciences, Glasgow University, UK; 2 Department of Physiology, Amsterdam UMC, Amsterdam Cardiovascular Sciences, Vrije Universiteit Amsterdam, The Netherlands; 3 Cardiovascular Research Centre, Icahn School of Medicine, New York, NY, USA; 4 Division of Applied Medicine, Pusan National University, Korea; 5 Department of Neuroscience, Ichan School of Medicine, New York, NY, USA; 6 INSERM, U1016, Institut Cochin, Paris, France; 7 Department of Clinical Biochemistry, Glasgow Royal Infirmary, UK; 8 Department of Physiology, University of Kentucky, Lexington, KY, USA; 9 Division of Cardiovasuclar Medicine, University of Kentucky, Lexington, KY, USA; 10 School of Health and Life Sciences, Glasgow Caledonian University, UK; 11 Flagship Pioneering, Cambridge, MA, USA; 12 Netherlands Heart Institute, Utrecht, The Netherlands

**Keywords:** heart failure, myocytes, myofilaments, SUMO, troponin I

## Abstract

Post-translational modification of the myofilament protein troponin I by phosphorylation is known to trigger functional changes that support enhanced contraction and relaxation of the heart. We report for the first time that human troponin I can also be modified by SUMOylation at lysine 177. Functionally, TnI SUMOylation is not a factor in the development of passive and maximal force generation in response to calcium, however this modification seems to act indirectly by preventing SUMOylation of other myofilament proteins to alter calcium sensitivity and cooperativity of myofilaments. Utilising a novel, custom SUMO site-specific antibody that recognises only the SUMOylated form of troponin I, we verify that this modification occurs in human heart and that it is upregulated during disease.

## Introduction

Troponin is a heterotrimeric myofilament protein which serves as the link between Ca^2+^ binding to the myofilament and cross-bridging of the thin and thick filaments, which ultimately leads to muscle contraction [1]. Each of the three subunits has a discrete function: troponin C (TnC) binds Ca^2+^, troponin I (TnI) has an inhibitory role and troponin T (TnT) binds tropomyosin, tethering the complex to the thin filament [2]. Post-translational modifications of cardiac excitation–contraction coupling proteins have been previously shown to be critical for the fine tuning of cardiac function. Particularly, phosphorylation of TnI at several sites results in functional changes at the myofilament [3]. Most well established is the functional role of Ser23/24 phosphorylation by protein kinase A upon beta-adrenergic receptor stimulation, which decreases myofilament Ca^2+^ sensitivity and contributes to enhanced contraction and relaxation [4–6]. It is well established that the phosphorylation level of TnI is altered in chronic cardiac disease, and likely is involved in its pathophysiology [7–9]. Although the phosphorylation events associated with TnI have been widely investigated, there is little evidence showing that TnI can be modified by other post-translational modifications. The present study is the first to show that TnI can be SUMOylated and that the modified protein functions differently within the myofilament.

SUMOylation is a reversible post-translational modification involving the covalent attachment of small ubiquitin-like modifier protein (SUMO) to a lysine residue of substrate protein. In mammals, three SUMO paralogues are expressed: SUMO1, SUMO2 and SUMO3. Despite their name, 11 kDa SUMO proteins share only 20% sequence homology with 8 kDa ubiquitin [10]. However, the enzymatic process for the addition of SUMO to a substrate is similar to the ubiquitination cascade [11]. The importance of SUMOylation in the heart has been shown by several knockout studies. Both global and cardiac-specific knockout of SUMO-1 in mice resulted in cardiac phenotypes including septal defects and sudden cardiac death while SUMO-2 knockout resulted in global developmental problems without any cardiac-specific effects [12–14]. This suggests that SUMO-1 has a prominent and vital role in the heart. As SUMOylation is reversible, alterations of the expression of various deSUMOylating enzymes, SENPs, have dramatic cardiac effects, with knockout of SENP2 causing embryonic death with significant cardiac phenotypes. Additionally, overexpression of SENP2 or SENP5 results in dilated cardiomyopathy, suggesting that uncontrolled de-SUMOylation is also detrimental [15,16]. In failing human myocardium, the expression of SUMO1 has been shown to be reduced suggesting that SUMOylation may have a protective role in human disease [12].

The role of SUMOylation in human heart disease has been investigated mainly in the context of SERCA2a SUMOylation. SUMOylation of SERCA2a enhances protein function and promotes its stability [12]. This was particularly beneficial in heart failure, which is characterised by a loss of function and expression of SERCA2a [17]. In fact, enhancement of SUMO-1 expression via gene therapy was shown to preserve SERCA2a expression and enhance cardiac function in a murine model of heart failure [18]. Furthermore, a small molecule activator of the SUMO E1 ligase (the first enzyme in the SUMOylation cascade) was shown to improve cardiac function and attenuate left ventricular remodelling in small and large animal models of heart failure [19]. To date, the SUMO modification of other important regulatory proteins involved in excitation–contraction coupling and the possible roles of this modification in disease have not been characterised.

Here we report for the first time that TnI can be SUMOylated at lysine 177. Investigations into the SUMOylation of specific substrates is often hampered by technical challenges in the detection of the modified protein [20]. To circumvent such problems, we developed a SUMO-TnI antibody, which specifically detects TnI that has been modified by SUMO at Lys177. Using our custom antibody, we report that SUMOylation of TnI is significantly upregulated in failing human myocardium compared to healthy myocardium. Additionally, we use transfection of SUMO-Null TNI mutants to show that this novel modification does not affect TNI stability or contractility of neonatal rat ventricular myocytes (NRVM) but instead, alters Ca^2+^ sensitivity of myofilaments under certain conditions.

## Results

### TnI is SUMOylated at K177

**Fig. 1.**
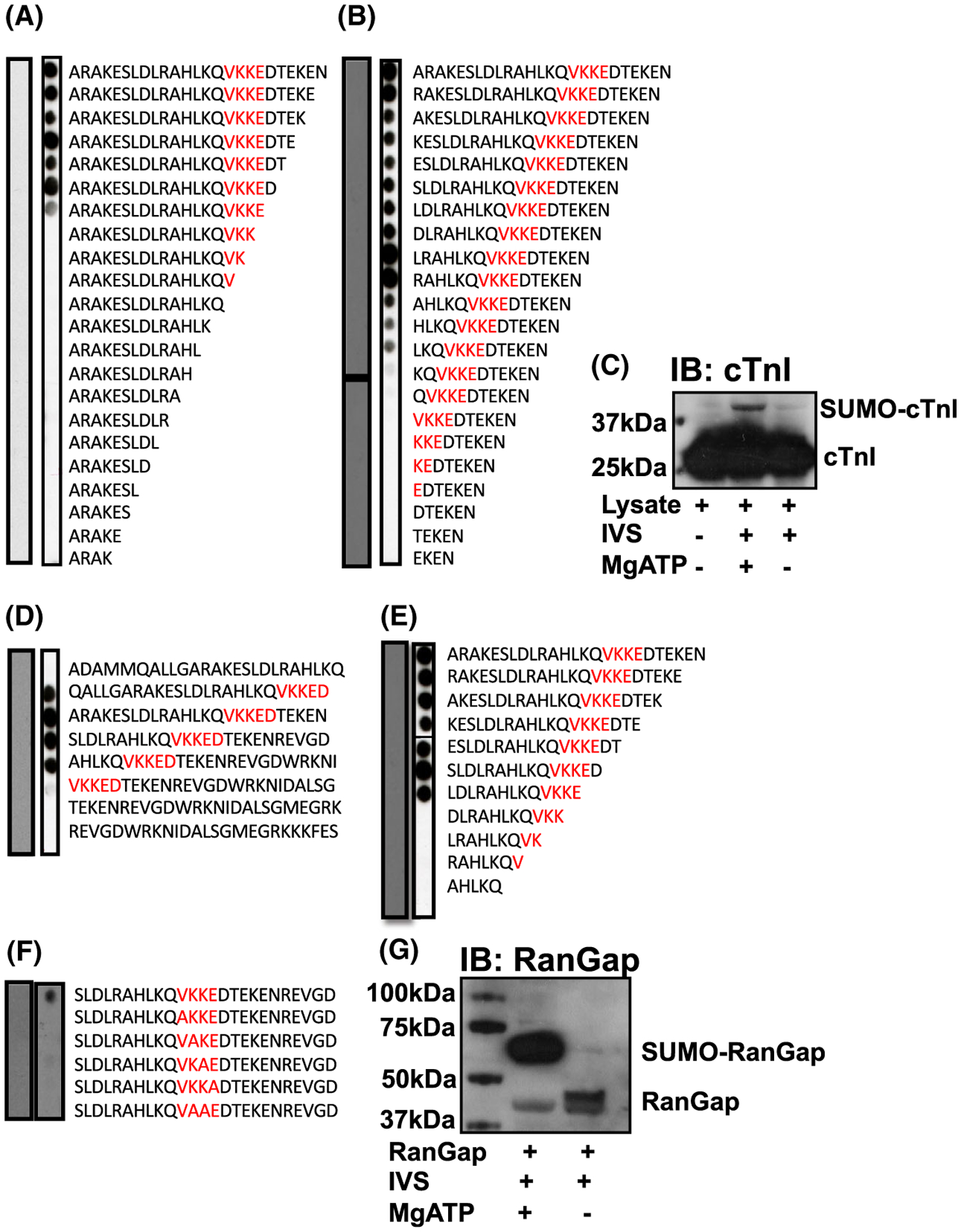
Delineation of a potential site of SUMOylation on TnI using peptide array technology. A 25-mer peptide the sequence that included potential SUMO-site (175VKKE-178) was (A) sequentially truncated at the C-terminal. (B) sequentially truncated at the N-terminal. Both arrays in (A and B) were treated with an invitro SUMOylation assay mix before being blotted for SUMO1. (C) Lysate extracted from NRVM was included in an *in vitro* SUMOylation assay. IVS = *in vitro* SUMOylation assay mix. Western blotting was used to identify TNI. (D) the potential SUMO motif (red) was sequentially shifted by five amino acids within peptide arrays containing the TNI sequence. (E) a 25-mer peptide with the amino acid sequence that included potential SUMO-site (175VKKE-178) was simultaneously truncated at N and C termini. (F) Essential amino acids within the putative SUMO motif (red) were sequentially substituted to alanine. Arrays in (D–F) were treated with an *in vitro* SUMOylation assay mix before being blotted for SUMO1. (G) A SUMOylation assay positive control showing SUMO modification of RanGap. IVS = *in vitro* SUMOYlation assay mix. Western blotting for RanGap.

As the SUMOylation of SERCA has been shown to be protective [21,22], we decided to search for putative SUMO-sites on other proteins that are vital to excitation–contraction coupling. Based on *in silico* analysis of TnI’s primary structure, we discovered a canonical four amino acid SUMOylation motif (hydrophobic, K, any amino acid, D/E) surrounding lysine 177. To assess the possibility of K177 being a *bona fide* SUMO site, *in vitro* SUMOylation assays were carried out on peptide arrays designed to include the region of TnI including K177 (Fig. 1). Immunoblotting SUMOylated peptide arrays of TNI sequence for SUMO1 allowed detection of positive spots in peptides where complete SUMO motifs (176-9VKKE) were present (Fig. 1D), but not when truncations (C-terminal: Fig. 1A, N-terminal: Fig. 1B and concomitant N- and C-truncation: Fig. 1E) positioned the accepting lysine close to the membrane support or abbreviated the motif. Furthermore, serial mutation of each residue within the SUMO motif to alanine attenuated SUMO1 modification (Fig. 1F), suggesting that each of the four amino acids within the motif is necessary for SUMOylation to occur. Peptide array experiments are inherently limited by the lack of secondary structure exhibited by synthesised 25-mer peptides, therefore, NRVM (neonatal rat ventricular myocytes) cell lysates which contain TnI at levels that are easy to detect by western blotting were included in *in vitro* SUMOylation assays. Comparison of assays including essential cofactor Mg ATP with assays in which Mg ATP was excluded revealed a bandshift when immunoblotted for TnI (Fig. 1C) suggesting that TNI could be modified by SUMO. The amount of SUMO-TNI (upper band) was small compared with the unmodified TNI (lower band) and this is likely as a consequence of deSUMOylating enzymes which are present in the NRVM cell lysates. Positive controls that demonstrate SUMOylation of a previously recognised substrate (Rangap1) validated the assay (Fig. 1G) Taken together, these experiments suggest that endogenous TnI can be SUMOylated and that this modification involves residue K177.

**Fig. 2.**
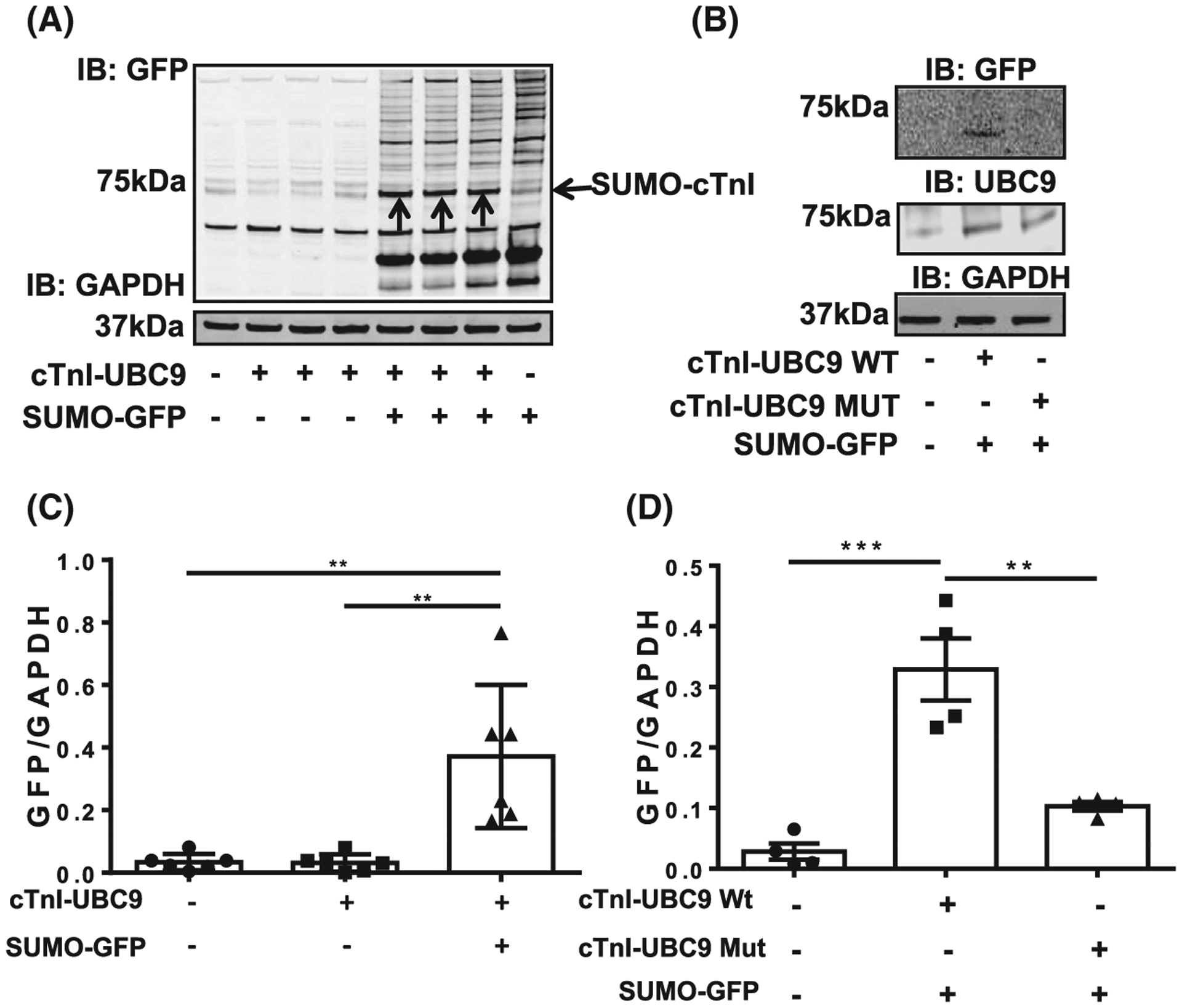
A fusion protein comprising of TNI conjugated to the SUMO E2 ligase UBC9 promotes TNI SUMOylation. (A) Lysates from HEK293 cells that had been transfected with TNI-UBC9 alone or co-transfected with SUMO1-GFP were probed for GFP and GAPDH using western blotting.(B) The wild type TNI-UBC9 fusion protein and a mutant (KK176/7-RR) were transfected into HEK293 cells along with SUMO1-GFP. Lysates were blotted for GFP, UBC9 and GAPDH. (C) Quantification of blot in (A). results are mean with standard errors, *n* = 6 **P = 0.01 one-way ANOVA with post hoc analysis: Turkey’s multiple comparison test. (D) Quantification of blot in (B) results are mean with standard errors, *n* = 4 **P = 0.01, **P = 0.001 vs. mock, one-way ANOVA with post hoc analysis: Tukey’s multiple comparison test.

To confirm that SUMOylation of TnI could occur in cells and that K177 was the SUMOylated lysine, UBC9 fusion-directed SUMOylation was employed [20]. In this technique the target protein is expressed as a fusion-protein with UBC9, the SUMO E2 ligase, and co-transfected into HEK-293 cells with SUMO-GFP. We hypothesised that the fusion of UBC9 to TnI would upregulate its SUMOylation and transfection with SUMO-GFP would allow for easy detection of the SUMOylated fusion protein. Co-transfection of HEK-293 cells with SUMO-GFP and the TNI-UBC9 fusion (but not either party alone) followed by western blotting for GFP resulted in detection of a band of 75 kDa (identified by arrows), suggesting that both fusion proteins (TNI-UBC9 and SUMO-GFP) were present at this weight (Fig. 2A, quantification Fig. 2C). Other bands exist at similar molecular weights that were recognised non-specifically by primary or secondary antibodies, however the SUMO-TNI can be clearly observed as prominent bands at 75 kDa (indicated with arrows). This data suggest that TnI can be SUMOylated in a cellular context. To confirm that the SUMOylation seen in Fig. 2A was specific to K177, a mutant version of TnI-UBC9 lacking the acceptor lysine (KK177-8RR) was co-transfected with SUMO-GFP. When these samples were immunoblotted, the ~ 75 kDa band was ablated, confirming that SUMOylation occurs within the 176-9VKKE SUMO motif (Fig. 2B, quantification Fig. 2D).

### Custom SUMO-TnI antibody specifically detects SUMOylated protein

**Fig. 3.**
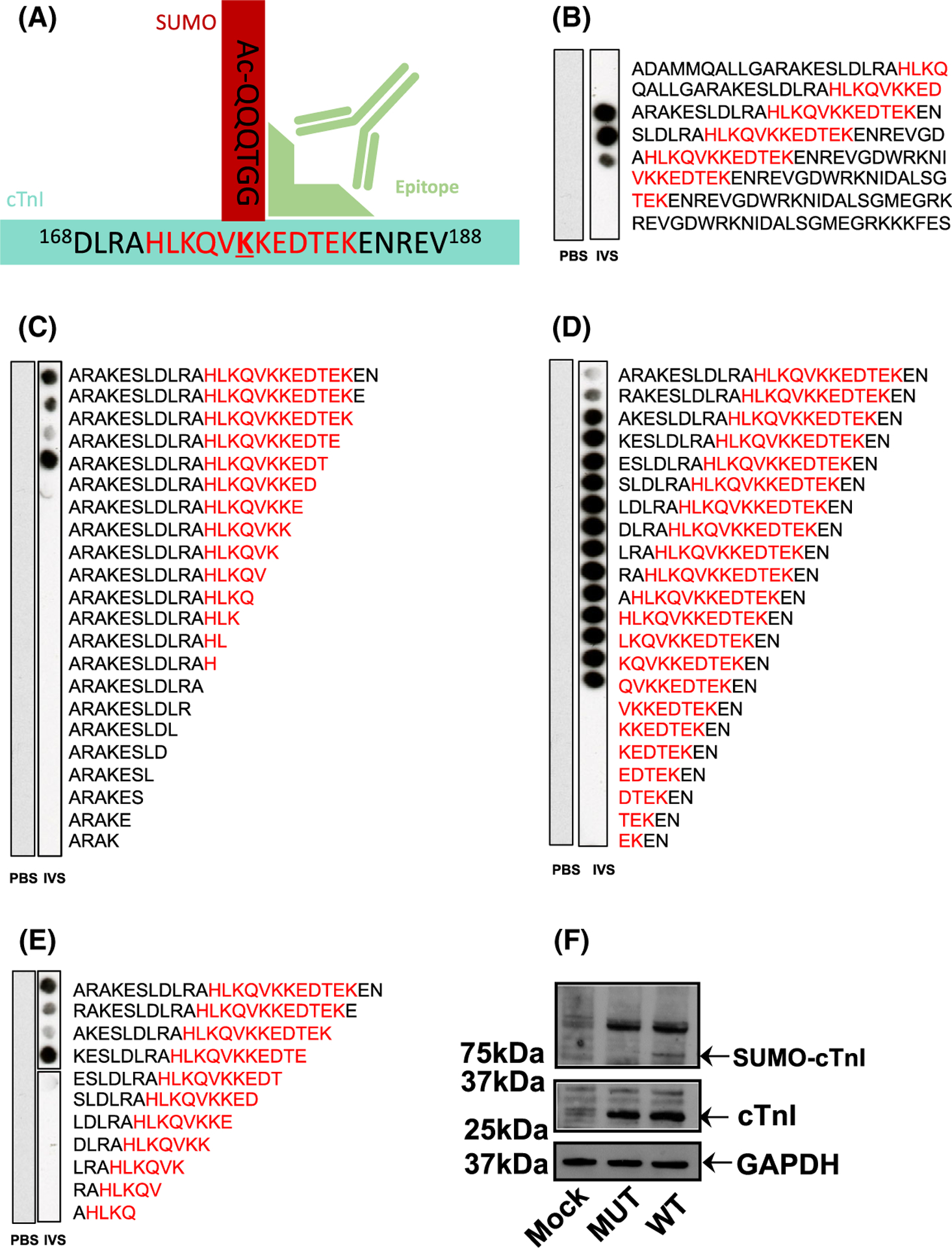
A custom antibody can specifically detect TNI SUMOylated within 175VKKE-178 motif. (A) Schematic representation of SUMO-TNI antibody illustrating that it was raised against the 12 amino acid sequence of TNI highlighted in red conjugated to SUMO1. The epitope that is recognised is shown in light green. (B) A scanning peptide array featuring the SUMOylated epitope (RED) was treated with an *in vitro* SUMOylation assay (IVS) or PBS and blotted with the custom SUMO-TNI antibody. Arrays in Figs (C–E) were treated in an identical fashion to that in (B). (C) A C-terminal truncation array starting with a 25-mer from TNI that contained the antibody epitope (D) a N-terminal truncation array starting with same peptide from (C). (E) A simultaneous N and C terminal truncation array starting with same peptide from (C). (F) Lysates from HEK293 cells transfected with TnI-HA WT or mutated (KK177/8-RR) TNI-HA or mock transfections were blotted with custom SUMO-TNI antibody and for GAPDH and TNI.

**Fig. 4.**
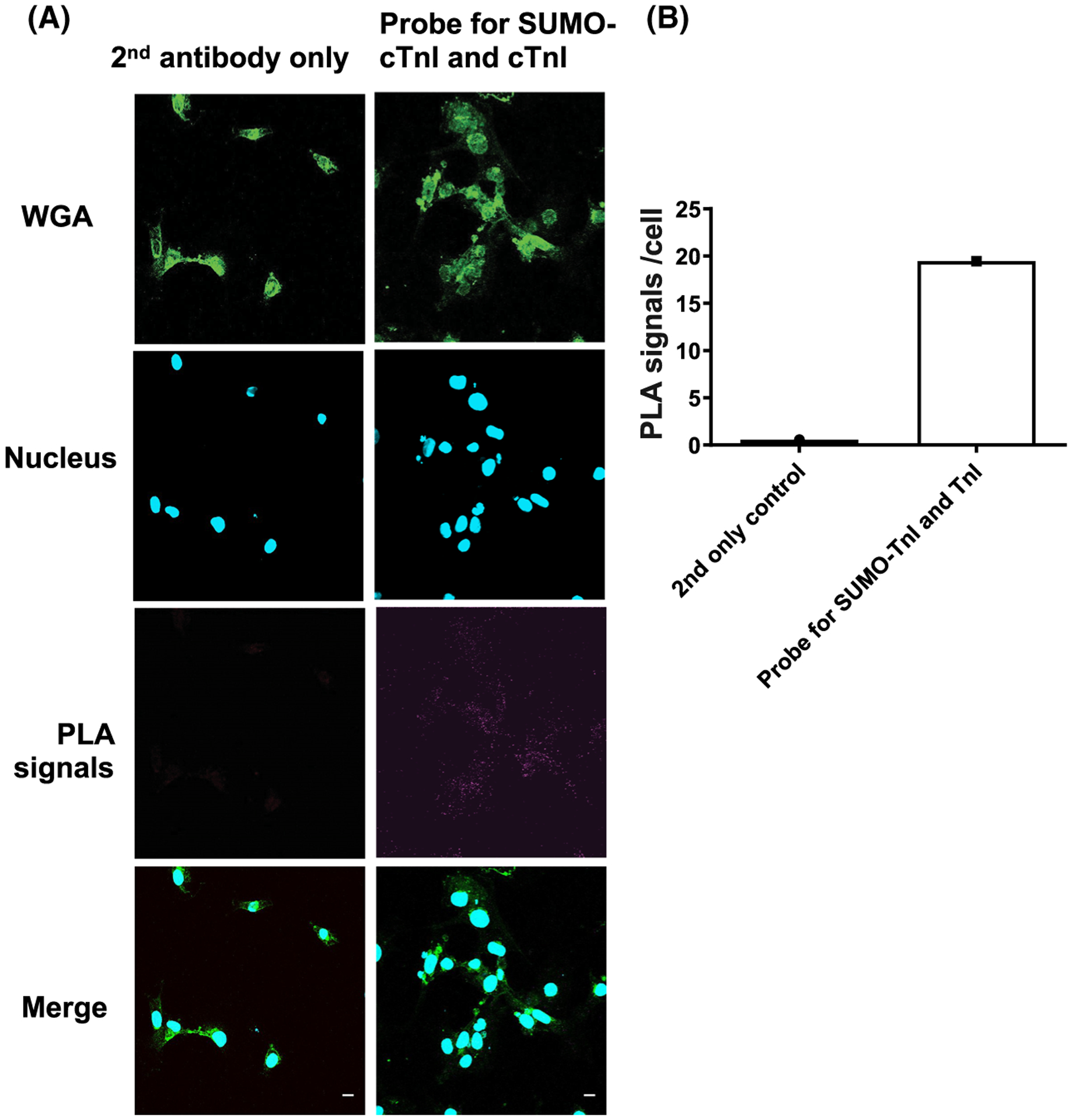
A custom SUMO-TNI antibody and commercially available TNI antibody co-localise in NRVMs. To show that the custom SUMO-TNI antibody and total TNI antibodies co-localise we used PLA. (A) NRVMs were fixed and stained and PLA analysis undertaken using a custom SUMO-TNI antibody and a TNI total antibody. (B) Quantification of the PLA signal detected in test slide vs. 2ndry antibody only control. Scale bars equal 10 μM.

Investigation of target protein SUMOylation is often impeded because detection can be technically difficult due to a low percentage of a specific SUMO substrate being modified at any one time and the avidity of SUMO-specific proteases. As SUMOylation often occurs at very low levels in cells, detection has previously relied on detection of faint SUMO band shifts following immunoprecipitation of the substrate [20]. To overcome these challenges, we designed and developed a custom antibody, which specifically detects SUMOylated TnI by interacting with an epitope that encompasses parts of both TnI and the covalently linked SUMO1 (Fig. 3A). To ensure that the antibody specifically detected the SUMOylated TnI epitope and not the unSUMOylated epitope, peptide arrays were subjected to *in vitro* SUMOylation assays and immunoblotted using the custom SUMO-TNI antibody. This resulted in positive spots when the full epitope (HLKQVKKEDTEK) was present and it had been subjected to SUMOylation (Fig. 3B). Additionally, N-terminal truncations (Fig. 3C), C-terminal truncations (Fig. 3D) and simultaneous N and C truncations (Fig. 3E) of sequences containing the antibody epitope confirmed that the C-terminal part of the epitope that contains the SUMO site (KKEDTEK) was most important for detection of TnI SUMOylation. These results support our idea that the recognition site required by the antibody (Fig. 3A) requires SUMO conjugation and the KKEDTEK motif. To make sure that our custom antibody could recognise SUMOylated TNI in a cellular context, the antibody was subsequently tested by immunoblotting HEK-293 lysates containing TnI-HA wildtype or TnI-HA mutant (ablated SUMOylation motif as previously described KK177–8RR). A band was detected at 75 kDa in the WT lane only, and not in untransfected HEK-293 cells or in cells transfected with TnI-HA mutant (Fig. 3F) suggesting that the novel antibody could recognise SUMOylated TnI in cells. It was hypothesised that the SUMOylated TnI band would be 12–25 kDa heavier than unmodified TnI. However, the larger than hypothesised protein can be explained by the likelihood that TnI is poly-SUMOylated. Each SUMO2 and SUMO3 protein contains a SUMOylation motif of its own, resulting in chains of SUMO proteins. SUMO1 does not contain a SUMOylation motif, and its addition terminates the chain [23,24]. To obtain further proof that our SUMO-TNI antibody could detect modified TnI in primary cultures, we used a PLA approach in NRVMs to show that a proportion of TnI in these cells colocalised with SUMO-TNI as detected by our antibody (Fig. 4A, quantification Fig. 4B). Collectively, our data suggests that TnI is modified by SUMOylation and that we have developed a SUMO-site-specific antibody that selectively recognises TnI in the SUMO-modified form.

### Functional role of TnI SUMOylation

**Fig. 5.**
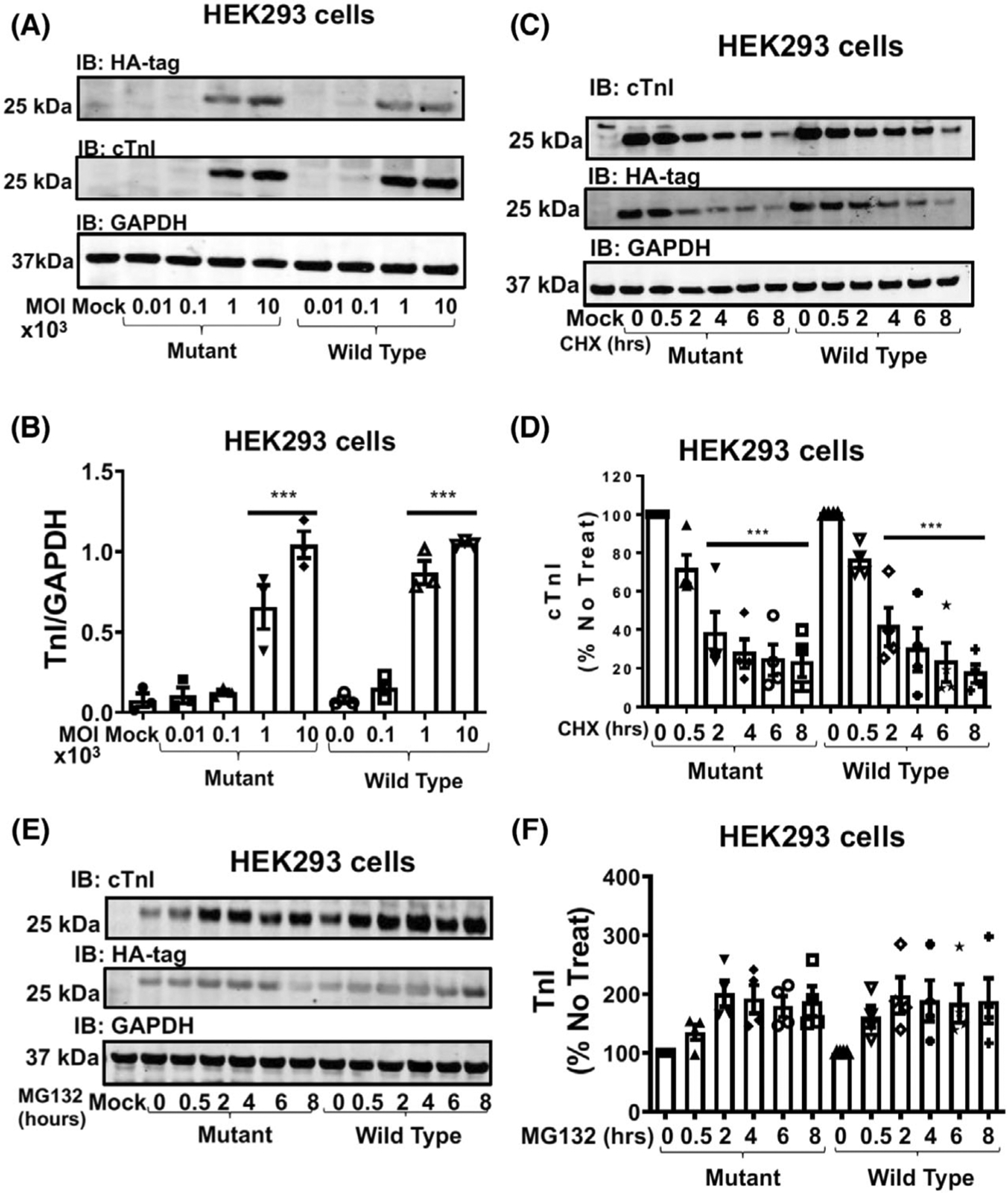
TNI SUMOylation does not affect TNI stability in HEK293 cells. (A) HEK293 cells transfected with different doses of virus encoding WT TNI-HA and mutant TNI-HA were blotted for HA, TNI and GAPDH. (B) Quantifications of normalised TNI (Wt and mutant) protein expression in HEK293 cells. Data expressed as mean plus standard error, *n* = 4. ***P < 0.001 vs. mock, one-way ANOVA with post hoc analysis: Tukey’s multiple comparison test. (C) HEK293 cells transfected with WT TNI-HA and mutant TNI-HA were treated with cycloheximide (50 μg·mL^−1^) for indicated times before lysates were prepared and blotted for HA, TNI and GAPDH. (D) Quantifications of normalised TNI (Wt and mutant) protein expression. Data displayed as mean plus standard error, *n* = 4. ***P = 0.001 vs. mutant 0 h and ***P < 0.001 vs. Wt 0 h. one-way ANOVA with post hoc analysis: Tukey’s multiple comparison test. (E) HEK293 cells transfected with WT TNI-HA and mutant TNI-HA were treated with the proteasome inhibitor MG132 (20 μM) for indicated times before lysates were prepared and blotted for HA, TNI and GAPDH. (F) Quantifications of normalised TNI (Wt and mutant) protein expression. Data displayed as mean plus standard error, *n* = 4. Data displayed as mean plus standard error, *n* = 4. One-way ANOVA with post hoc analysis: Tukey’s multiple comparison test.

As this is the first study to report SUMO-modification of TnI and the site at which it occurs, our next step was to investigate the functional consequence of TnI SUMOylation. In an attempt to discover a role for this modification, we constructed adenoviruses that contained wild type TnI, tagged with an HA tag (TnI-HA WT) and a mutated form (KK177-RR) with HA tag (TnI-HA MUT) of TnI that had previously been shown to preclude SUMO modification (Figs 1F, 2B, and 3F). As the SUMOylation of SERCA protected the protein against proteosomal degradation [12] we were keen to see if the half-life of TnI was similarly affected. Firstly, we were able to get robust over-expression of both WT and MUT TnI forms in HEK293 cells to allow us to undertake stability tests (Fig. 5A). Importantly, the WT and MUT TnI expressed at the same level according to dose (Fig. 5B). In this model system, protein levels of TnI were significantly reduced after 2 h of cycloheximide treatment (Fig. 5C) but this rapid reduction in protein level was similar for the WT and MUT TnI (Fig. 5D). As the short half-life of TnI in HEK293 cells suggested turnover by the 26S proteasome, we repeated the experiment with MG132, a proteasome inhibitor. As expected, blockade of the proteasome resulted in a rapid upregulation of TnI protein (Fig. 5E) but again the WT and MUT TnI showed an identical trend (Fig. 5F) suggesting that SUMOylation of TnI did not affect turn-over of the myofilament protein when transfected into HEK293 cells.

**Fig. 6.**
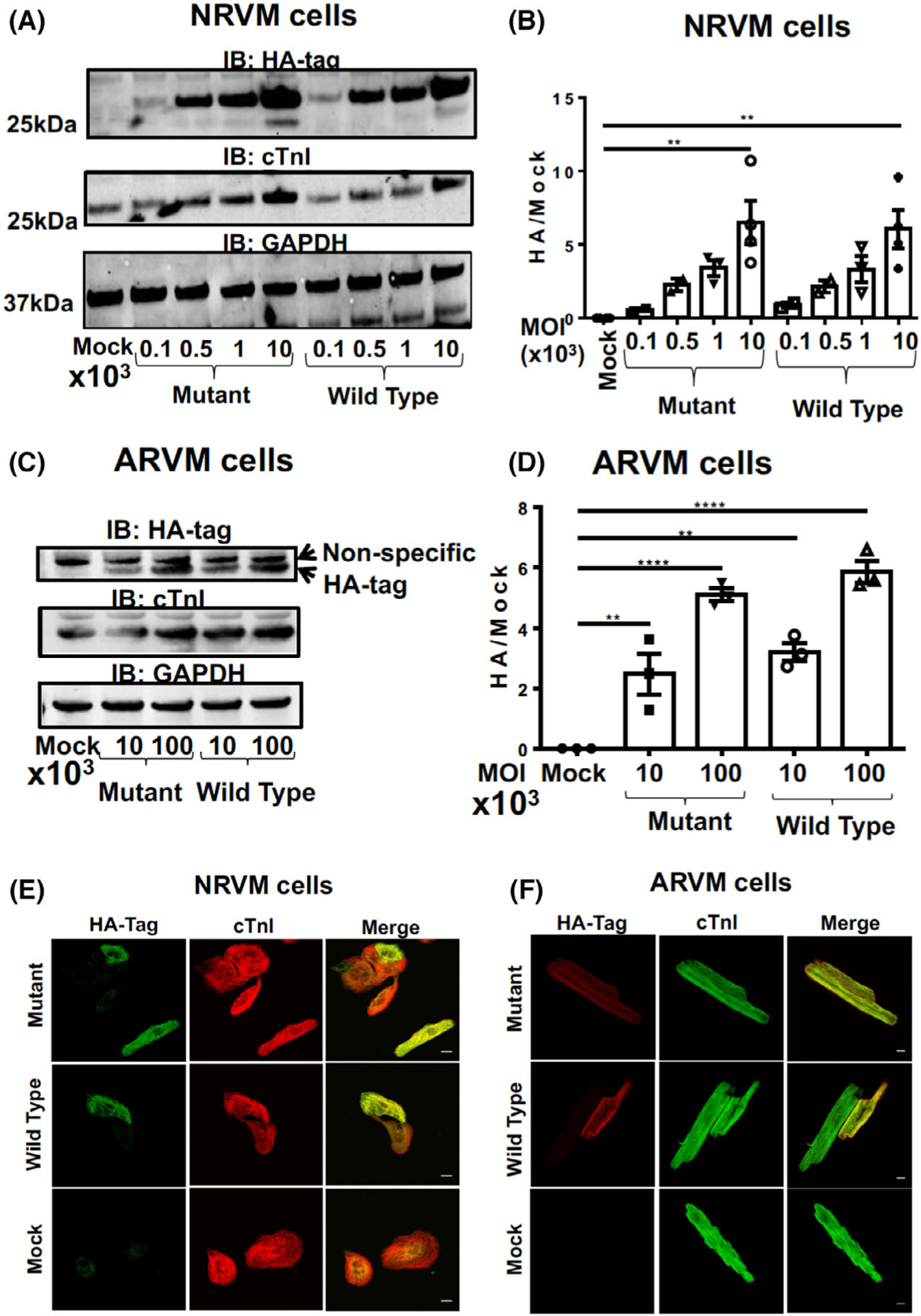
Primary ventricular myocytes virally transfected with wild type and SUMO mutant forms of TNI express the constructs at the same level (A) NRVM cells were infected with viruses expressing mutant and wild type TNI-HA in increasing doses before lysates were harvested and blotted for HA, TNI and GAPDH. (B) quantification of overexpression of WT TNI-HA and mutant TNI-HA with increasing dose from blots depicted in (A) results represented as mean with standard errors, *n* = 4.**P = 0.001 vs. mock, one-way ANOVA with post hoc analysis: Tukey’s multiple comparison test. (C) ARVM cells were infected with viruses expressing mutant and wild type TNI-HA in increasing doses before lysates were harvested and blotted for HA, TNI and GAPDH. (D) quantification of overexpression of WT TNI-HA and mutant TNI-HA with increasing dose from blots depicted in a. results represented as mean with standard errors, *n* = 4.**P < 0.001 vs. mock, ***P < 0.0001 vs. mock, one-way ANOVA with post hoc analysis: Tukey’s multiple comparison test. (E) NRVM cells infected with WT TNI-HA and mutant TNI-HA (MOI 10^2^) were fixed and stained with antibodies against HA and TNI which were labelled with AlexaFluor 633 and 488 respectively. Controls were mock transfected NRVMs and NRVMs stained with 2ndry only. (F) ARVM cells were treated in exactly the same way as in (E). Scale bar equals 10 μM.

**Fig. 7.**
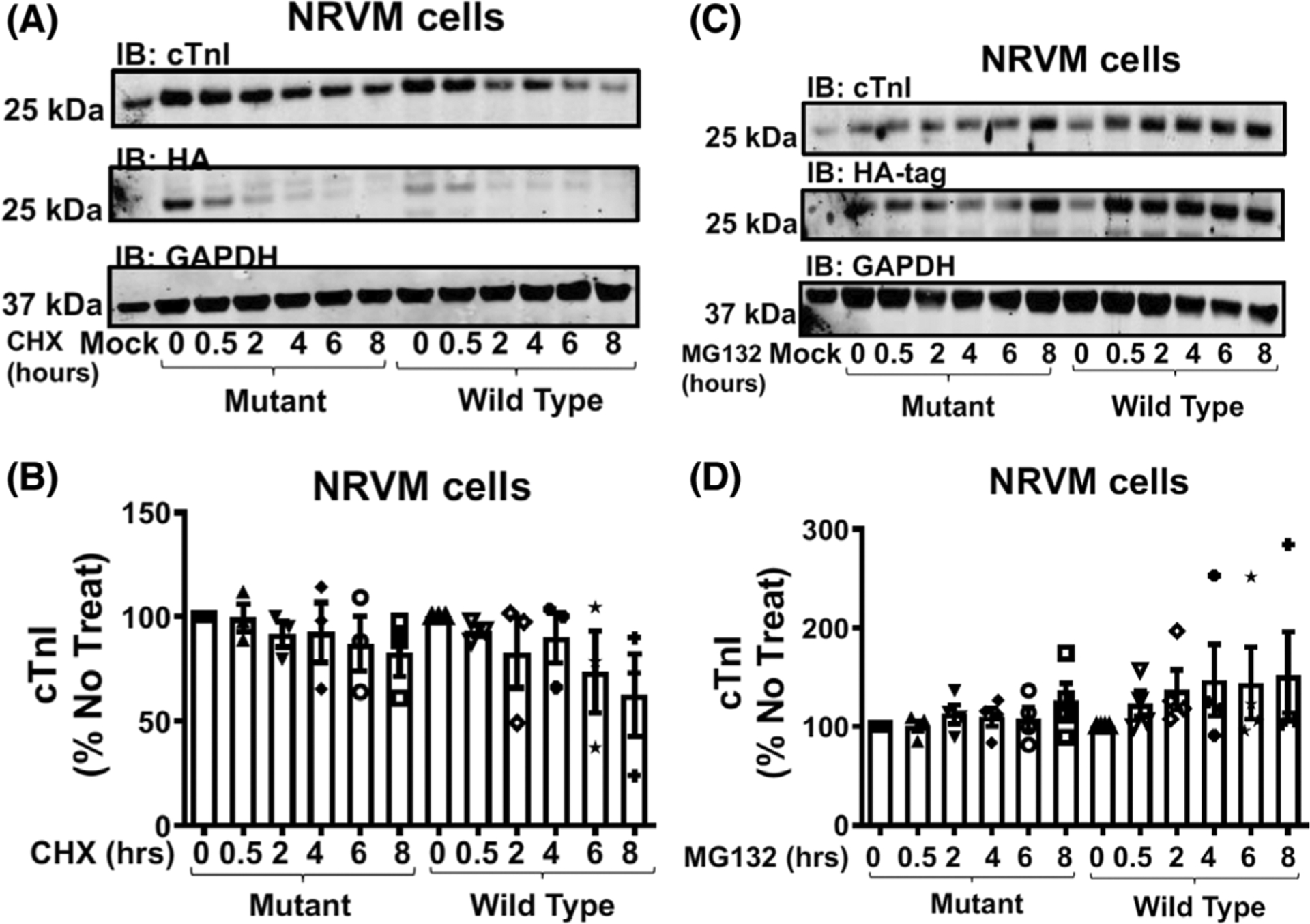
TNI SUMOylation does not affect TNI stability in NRVM cells. (A) NRVM cells transfected with WT TNI-HA and mutant TNI-HA were treated with cycloheximide (50 μg·mL^−1^) for indicated times before lysates were prepared and blotted for HA, TNI and GAPDH. (B) Quantifications of normalised TNI (Wt and mutant) protein expression. Data displayed as mean plus standard error, *n* = 4., one-way ANOVA with post hoc analysis: Tukey’s multiple comparison test. (C) NRVM cells transfected with WT TNI-HA and mutant TNI-HA were treated with the proteasome inhibitor MG132 (20 μM) for indicated times before lysates were prepared and blotted for HA, TNI and GAPDH. (D) Quantifications of normalised TNI (Wt and mutant) protein expression. Data displayed as mean plus standard error, *n* = 4. Data displayed as mean plus standard error, *n* = 4. One-way ANOVA with post hoc analysis: Tukey’s multiple comparison test.

To allow us to evaluate physiological changes that result from a SUMO-null version of TnI, we infected both NRVMs (Fig. 6A) and ARVMs (Fig. 6C) with the viruses containing TnI-HA WT and TnI-HA MUT. Once again, repeatable and robust over-expression of both TnI forms was evident in both cell types with expression levels for TnI-HA WT equalling that of TnI-HA Mut (Fig. 6B,D). Examination under a confocal microscope confirmed that both virally transfected WT and MUT TnI localised correctly to the myofilament compartment in both NRVMs (Fig. 6E) and ARVMs (Fig. 6F). As with TnI expressed in HEK293 cells (Fig. 5), the half-life (Fig. 7A,B) and susceptibility to turnover by the proteasome (Fig. 7C,D) was similar between WT and MUT TnI when over expressed in NRVMs suggesting that SUMOylation does not affect TnI stability in physiologically-relevant primary cells.

**Fig. 8.**
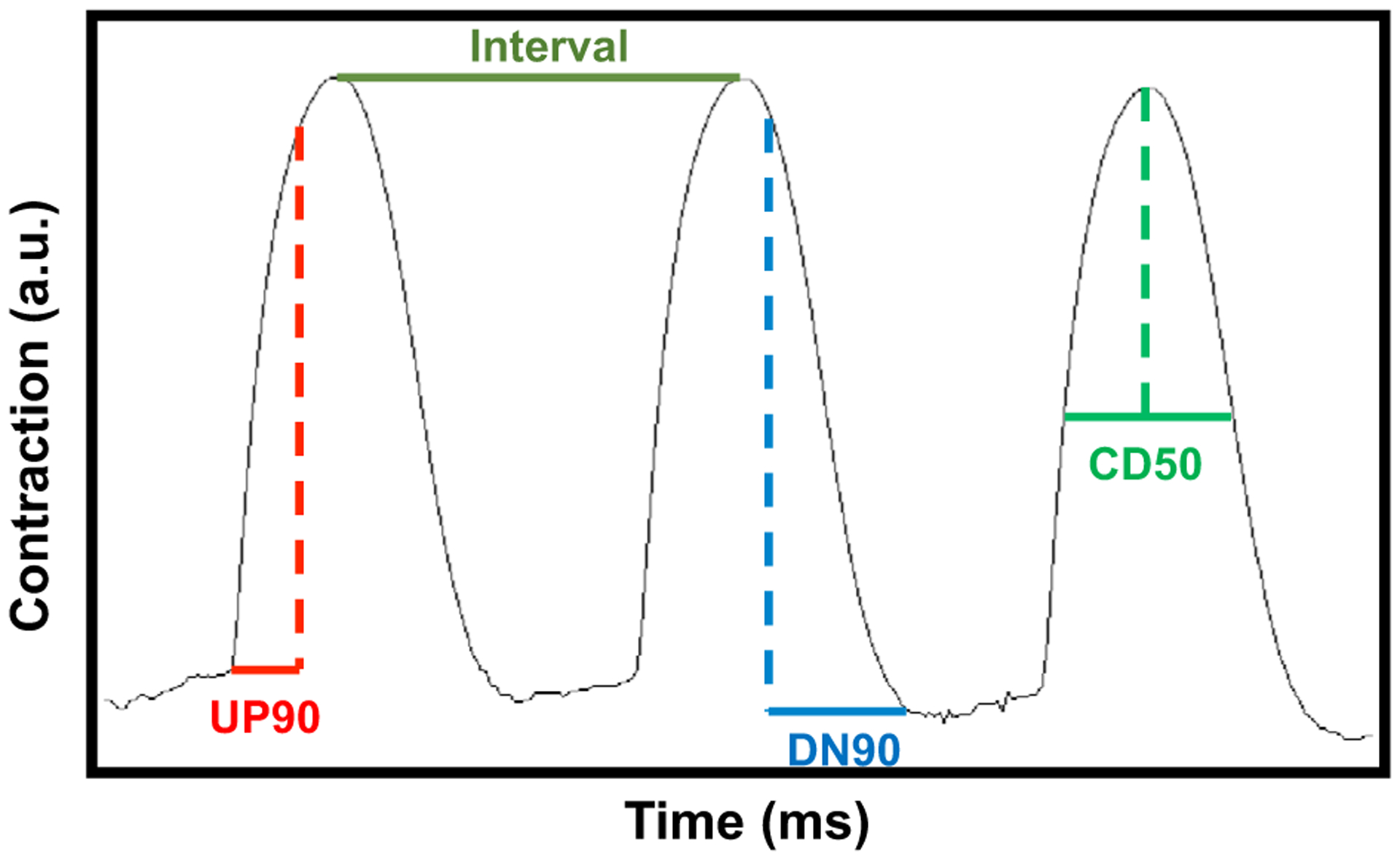
Schematic representation of contraction and relaxation parameters. Contraction traces generated by musclemotion software. Interval (in orange) or time between contractions was used to evaluate how well NRVM monolayers adhere to pacing. UP90 (in red) is a measure of time to contract and represents time rom baseline to 90% of the peak, DOWN90 (in blue) measures time to relax and is calculated as time from 90% peak back to baseline. Finally, contraction duration at 50% (in green) is recorded as time between 50% of upstroke vs. 50% of DOWN stroke.

**Fig. 9.**
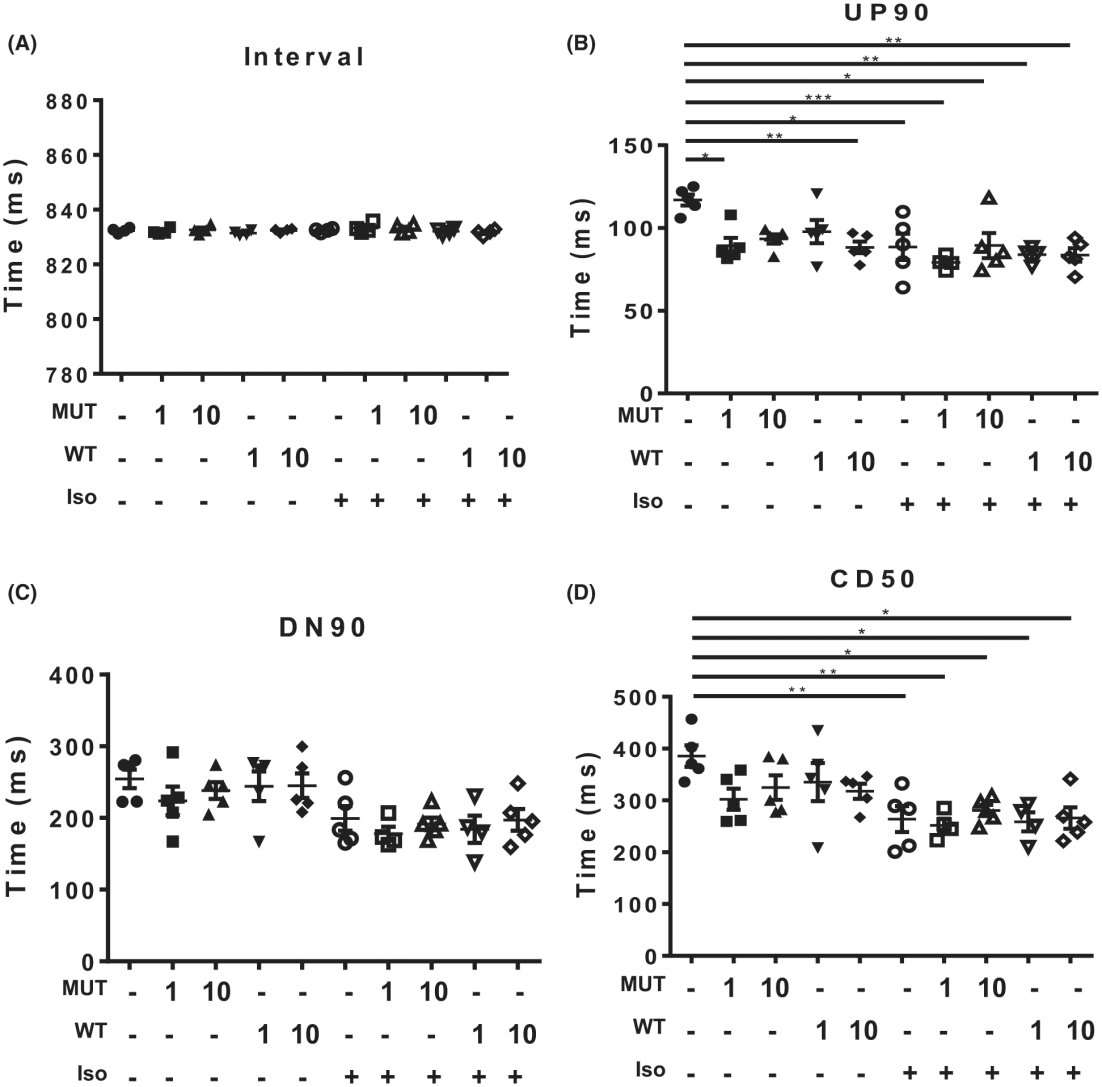
Testing the involvement of SUMO-TNI in contractility and calcium sensitivity using virally transfected myocytes. Using CellOPTIQ, NRVMS (mock transfection or transfected with 1 or 10 MOI) were subjected to pacing at 1.2 Hz and images were captured at 100 frames per second for 8 s. Each sample was measured at baseline and following 10 min of 100 nM isoproterenol treatment. (A) The interval time between contractions was measured to ensure NRVM monolayers conformed to exogenous pacing. (B) UP90, the time from baseline to 90% of peak upstroke was measured as an estimate of time to contract. (C) DOWN90, the time from 90% contraction after the peak back DOWN to baseline was determined to evaluate the relaxation time. (D) CD50, the time from 50% upstroke to 50% down stroke was measured to take both contraction and relaxation into account. Each data point in all graphs is representative on the mean value from one experimental day, *n* = 5 experimental days. Each mean was calculated from between 6 and 10 recordings of 8 to 10 contractions per condition. *P = 0.05 vs. mock and **P < 0.01, and ***P < 0.001. To investigate myofilament function, force measurements were performed at a sarcomere length of 2.2 μm in ARVMs overexpressing WT TNI-HA and mutant TNI-HA which were incubated with N106 for 24 h and skinned. (E) The calcium concentration resulting in half maximal force (EC50) and (F) steepness of the dose response curve between Ca^2+^ and force were determined as hill slope. Data represents mean plus standard error, *n* = 12 cells from three isolations for untreated samples; *n* = 8 cells from two isolations for N106 treated samples. *P < 0.05 vs. mock + N106 and **P < 0.01 vs. WT + N106. (G) in addition, passive force (*F*_passive_) and (H) maximum force (*F*_max_) were determined *P < 0.05 vs. mock and $*P* < 0.05 vs. MUT. All analysis used one-way ANOVA with post hoc analysis: Tukey’s multiple comparison test.

To determine whether SUMOylation of TnI affects the contraction dynamics of cardiac myocytes, we used virally transfected NRVM monolayers under baseline conditions or under stimulation with isoproterenol in conjunction with CellOPTIQ technology. This system allows accurate measurement of contractility in live cells [25] (outlined in Fig. 8). We note that NRVMs may express slow skeletal forms of TNI and adopted an overexpression approach to reduce the influence of endogenous isoforms. All NRVM cells were paced at1.2 Hz and showed consistent contraction rates under all of the different conditions (Fig. 9A). Measurement of the time to 90% of peak contraction (UP90) was not significantly different between MUT TnI and WT TnI transfected cells at any of the transfection rates, however there was a significant reduction of UP90 when the mock cells were compared to WT MOI 10^4^ and MUT MOI 10^3^ (Fig. 9B). As expected, isoprenaline treatment caused a significant decrease in UP90 in control (mock) cells and a trend to decreased UP90 in all cells, however statistical differences between WT TnI and MUT TnI transfected cells were not detected suggesting that TnI SUMOylation did not alter contraction times (Fig. 9B). Data for DOWN90 (time to 90% of relaxation) also indicated that SUMOylation of TnI did not affect lusitropy as transfected cells had similar relaxation times to mock controls (Fig. 9C). Anticipated positive lusitropic effects were triggered by isoprenaline in all cells (Fig. 9C). In a similar fashion to UP90 and DOWN90, contraction duration (CD50) was also unchanged when WT TnI and MUT TnI transfected cells were compared (Fig. 9D).

**Fig. 10.**
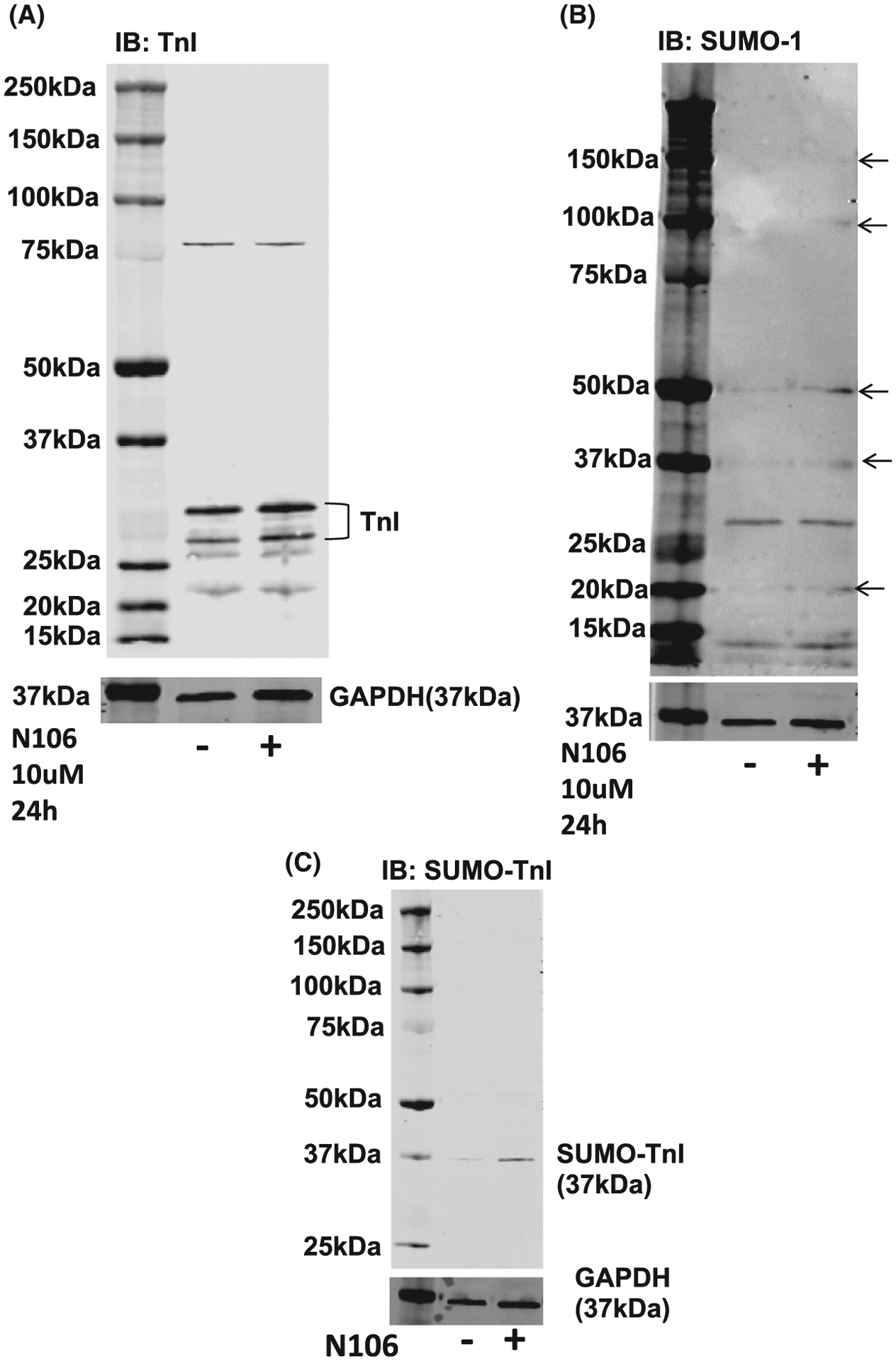
N106 treatment activates the SUMO cascade and enhances SUMO-TNI in ARVMS. (A) Treatment of ARVMs with N106 (10 μM for 24 h) did not affect TNI expression. (B) Treatment of ARVMs with N106 (10 μM for 24 h) upregulated SUMOylation of cellular SUMO substrates (indicated by arrows). Figure representative of *n* = 3. (C) Treatment of ARVMs with N106 (10 μM for 24 h) upregulated SUMOylation of TNI as measured with the custom SUMO-TNI antibody. Figure representative of *n* = 3.

**Fig. 11.**
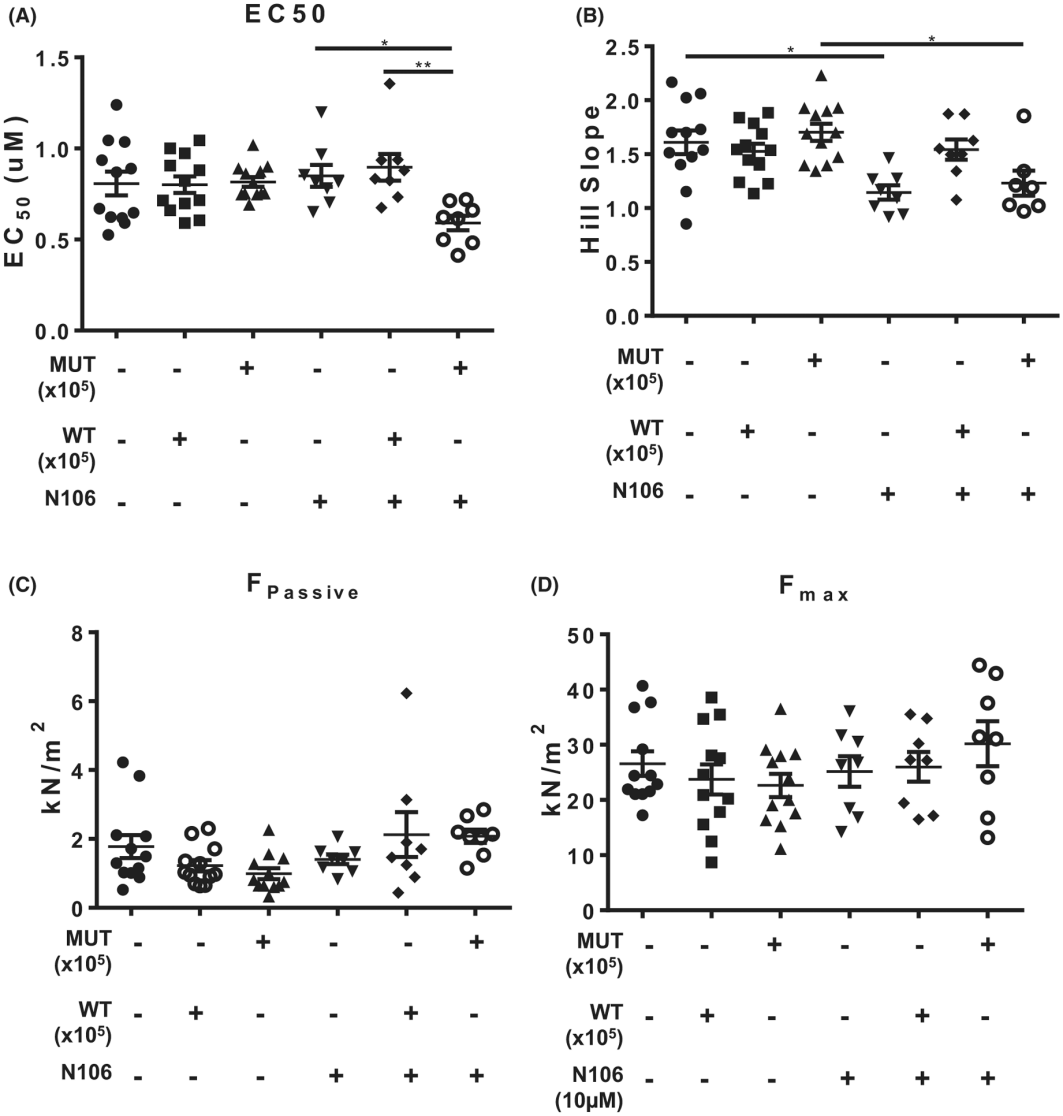
Evaluating N106 treatment in ARVMS. To investigate myofilament function, force measurements were performed at a sarcomere length of 2.2 μm in ARVMs overexpressing WT TNI-HA and mutant TNI-HA which were incubated with N106 for 24 h and skinned. (A) The calcium concentration resulting in half maximal force (EC50) and (B) steepness of the dose response curve between Ca^2+^ and force were determined as hill slope. Data represents mean plus standard error, *n* = 12 cells from three isolations for untreated samples; *n* = 8 cells from two isolations for N106 treated samples. *P < 0.05 vs. mock + N106 and **P < 0.01 vs. WT + N106. (C) In addition, passive force (*F*_passive_) and (D) maximum force (*F*_max_) were determined *P < 0.05 vs. mock and $*P* < 0.05 vs. MUT. All analysis used one-way ANOVA with post hoc analysis: Tukey’s multiple comparison test.

Although SUMOylation of TnI did not impinge on NRVM contractility, we were keen to determine if there was a significant change in myofilament functional properties when comparing WT TnI and MUT TnI. In these experiments we used membrane-permeabilised ARVM muscle fibres [26] in conjunction with N106 treatment. Compound N106 has previously been shown to promote SUMOylation in cardiac systems by activating SUMO E1 enzymes [19]. In line with previous findings, we observed that N106 upregulated global SUMOylation (Fig. 10B: arrows) but did not affect total TNI levels in ARVMs (Fig. 10A). We also measured an enhancement of SUMO-TNI in ARVMs evaluated with the custom SUMO-TNI antibody (Fig. 10C). In light of this, we hypothesised that activation of the SUMO cascade would alter Ca^2+^ sensitivity of the myofilaments of WT TnI transfected cells but not MUT TnI, however, under basal conditions mock, WT TnI and MUT TnI ARVM cells exhibited similar EC50 values (Fig. 11A). Paradoxically, Ca^2+^ sensitivity was significantly increased in cells over-expressing MUT TnI where global SUMOylation had been induced with N106 (Fig. 11A, last lane). Interestingly, upregulation of SUMOylation with N106 caused a significant reduction in the Hill slope (Fig. 11B), which reflects the steepness of the calcium-force dose response relations, from mock and MUT TnI transfections but not from skinned cells transfected with WT TnI (Fig. 11B). When we compared maximum (*F*_max_; Fig. 11C) and passive (*F*_Passive_) force (Fig. 11D) measurements at a sarcomere length of 2.2 μm, we could not detect any significant differences between the WT TnI and MUT TnI suggesting that TnI SUMOylation was not a factor in the development of passive and maximal force generation in response to calcium in the skinned cells.

### SUMOylated TnI is increased in heart failure

**Fig. 12.**
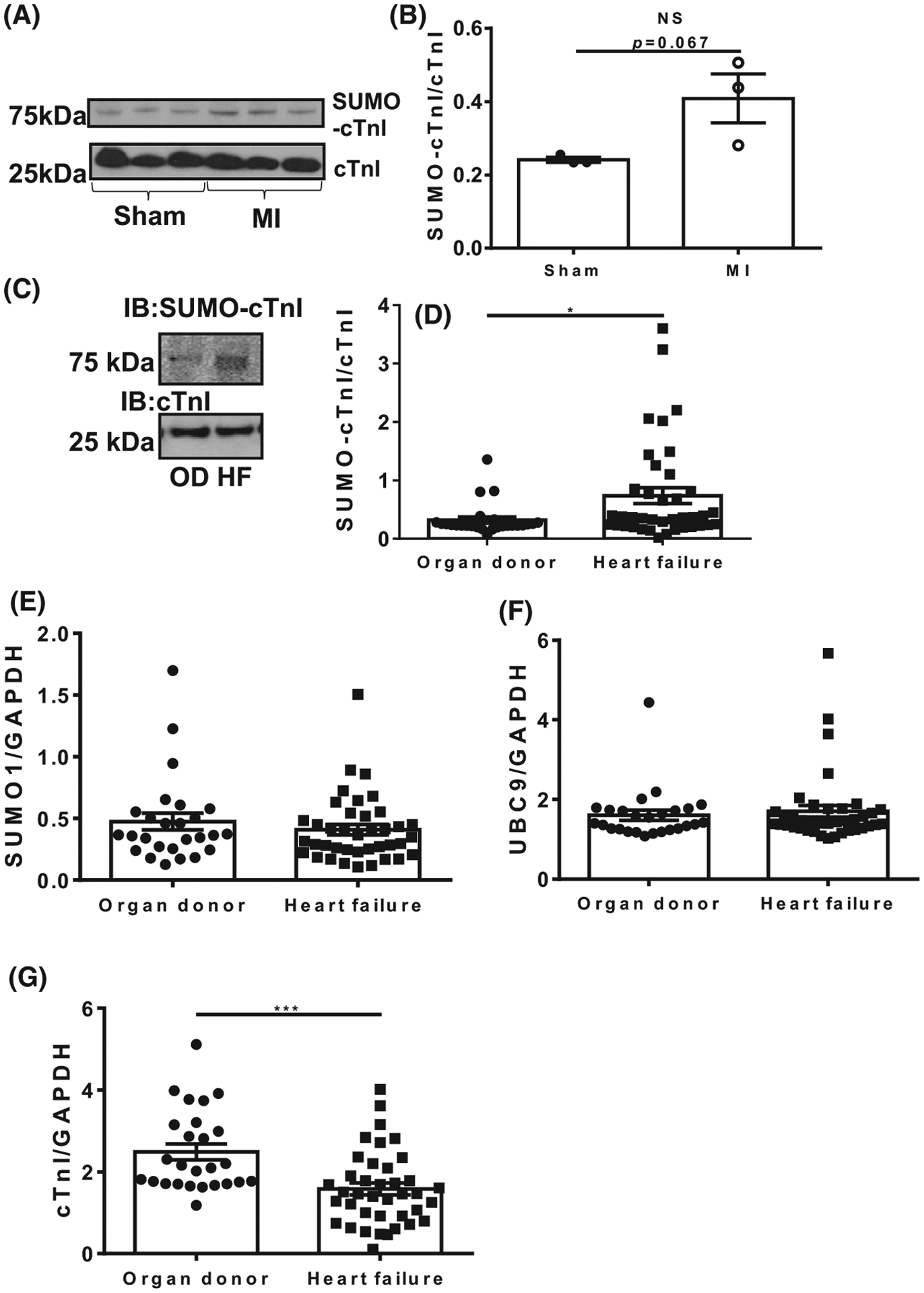
SUMOylation of TnI is increased during heart disease. (A) Heart samples from porcine LAD artery balloon occlusion models of MI were blotted for TNI and SUMO-TNI using the custom antibody. (B) Results represented as mean plus standard error, *n* = 3, unpaired student’s t-test. (C) Human heart samples from healthy organ donors (O.D.) or from diseased hearts were blotted for TnI and SUMO-TNI using the custom antibody. (D) Data are displayed as mean plus standard error, *n* = 26 healthy and *n* = 38 diseased. ***P = 0.001, student’s unpaired t-test. (E) Quantification of total SUMO1 in human heart samples using western blotting. (F) Quantification of total; UBC9 from human heart samples using western blotting. (G) Quantification of total troponin I in heart failure samples compared to housekeeping gene GAPDH. Data are displayed as mean plus standard error, *n* = 26 healthy and *n* = 38 diseased. ***P = 0.001, student’s unpaired t-test.

Based on previously published data showing that SUMO1 expression and SUMOylated SERCA2a are decreased in the failing myocardium, we hypothesised that the SUMOylated TnI would also be altered in heart failure [12]. The successful development and testing of the SUMO-TnI specific antibody allowed the relative SUMOylation levels to be directly compared in relevant biological samples. Firstly, we evaluated levels of SUMOylated TnI in tissue samples from a porcine model of myocardial infarction (MI) in which pigs were subjected to left anterior descending artery balloon occlusion. The level of SUMOylated TnI was higher in MI compared to Sham samples (Fig. 12A,B).

**Fig. 13.**
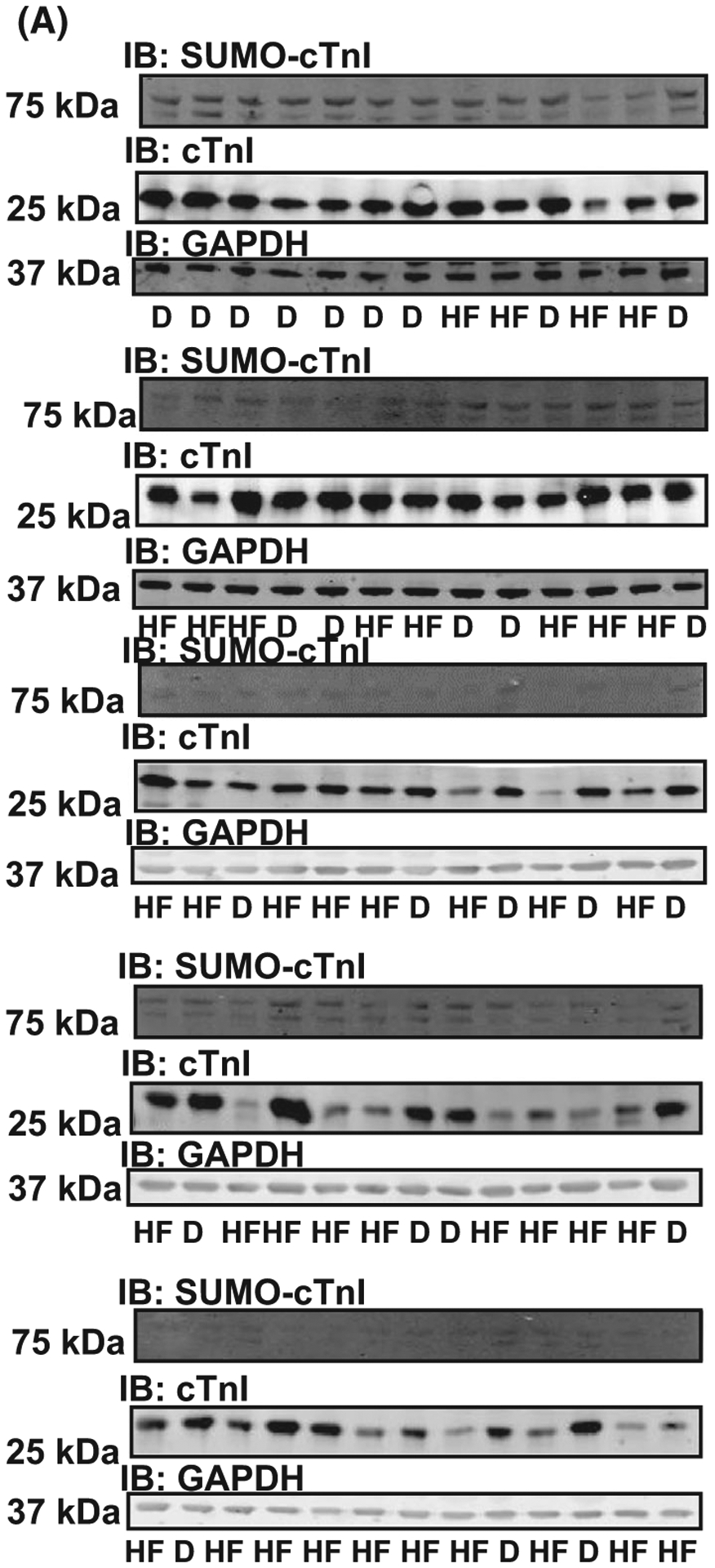
Probing human heart failure samples for SUMO-troponin I, troponin I and GAPDH. (A) Examples of western blots from all of human samples used in Table 1, HF, heart failure and D, donated heart). Mean data expressed in Fig. 12D.

In human heart samples outlined in Table 1, there was a significant increase in the level of SUMOylated TnI in failing myocardium compared to healthy myocardium from organ donor samples (Fig. 12C,D). Levels of SUMO1, UBC9 and TNI were similar between control and disease samples (Fig. 12E–G) suggesting that TnI SUMOylation was a highly specific event that was not simply driven by a global upregulation of the general SUMOylation machinery. Western blots depicting all of the human heart samples tested for TNI and SUMO TNI are depicted in Fig. 13.

## Discussion

Recent evidence suggests that SUMOylation of cardiac proteins is a disease-related mechanism [21] that can be detected during oxidative stress [27], cardiac hypertrophy [28], Ischaemia reperfusion (I/R) injury [29], cardiac fibrosis [30], hypertension [31] and arrhythmia [32]. Although some of the SUMO substrates that are modified during these diseases have been identified, for example, NaV1.5 for arrhythmia [32], PPARgamma for I/R [33] and PML for cardiac fibrosis [34], reports of SUMO modification of calcium handling proteins involved in excitation-contraction coupling are restricted to the pioneering work of Hajjar and co-workers on SERCA (reviewed in [22]). SUMOylation of SERCA (promoted by SUMO1 gene transfer) protected SERCA from natural degradation (a common feature of heart failure) and maintained sarcoplasmic reticulum calcium cycling, improving velocity of muscle contraction and relaxation, increased cell shortening, enhanced calcium re-uptake and survival. The overall inotropic effect triggered by SERCA SUMOylation mimicked effects observed after SERCA2 deficient failing hearts were ‘rescued’ by SERCA2 gene-transfer. Interestingly, SUMO1 gene therapy was not effective in cells where SERCA2 was absent, suggesting that SERCA2 is the most important SUMO substrate [18] for cardioprotection. Protective effects in heart failure models that promote cardiac contractility have also been recorded in animals that have their SUMO signalling networks boosted by pharmacological activation of SUMO E1 enzymes [19] or inhibition of SUMO1 suppressing miRs [28]. These methods along with SUMO gene transfer have the potential to drive concomitant SUMO modification of a variety of cardiac substrates and we were compelled to look for other calcium signalling proteins that may also be adapted by the covalent ligation of SUMO proteins. A search for SUMO motifs in this diverse group of proteins uncovered a canonical SUMO sequence in TnI (^176^VKKE^179^) and our biochemical data (Figs 1, 2 and 3) strongly suggests, for the first time, that this novel modification is not only possible but that it happens in mammalian cells. Previous work on TnI orthologues in Drosophila have identified SUMO modifications that are important in embryogenesis [35]. However, as the human and Drosophila sequences share only 22% sequence identity, it is difficulty to translate this observation into the human context.

Much effort has gone into researching the post-translational modification of TnI, but has concentrated on the protein’s various phosphorylation sites [3]. Specifically, phosphorylation of the dual serine site Ser23/24 has been shown to contribute to the β-adrenergic receptor stimulation induced positive lusitropy and inotropy, enhancing cardiac contractility [36,37]. Phosphorylation of this site is also reduced in chronic heart disease, prompting investigation of the functional role of this modification [38]. We have developed a SUMO-site-specific antibody for the detection of TNI (Fig. 3) to show that in both tissues samples from a porcine model of MI and in human samples (Fig. 12) the levels of SUMO-TNI are increased. Unlike in the case of SERCA where SUMO modification enhances the stability of the protein and constitutes a protective mechanism, SUMO-null TNI mutants had the same half-life as WT constructs (Figs 5 and 7), suggesting that the functional role of TnI SUMOylation does not hinge on the balance between TnI translation and protein degradation by the proteosome. Clues to why SUMOylated TnI is upregulated in heart disease may be deduced from appreciation of the functional role of the modification.

In searching for a physiological function for SUMOylation of TnI we utilised viral overexpression of WT and SUMO-null TNI (Figs 9 and 11) in NRVM cells and evaluated contractility and Ca^2+^ sensitivity of the myofilaments. There were no discernable changes in contractility or lusitrophy, however development of force in response to Ca^2+^ was increased and cooperativity of interacting cross-bridges was decreased (as assessed by Hill Slope) in myofilaments over-expressing SUMO-null TnI (but not wild type) following activation of the SUMO cascade with N106 (Fig. 11). While no direct effect on calcium-activated myofilament properties were observed, SUMOylated TNI may induce structural changes in the thin filament, and underlie the changes observed in the MUT TNI cells in which global SUMOylation was induced by N106. Recent electron cryomicroscopy studies have shed light on the structure of the thin filament composed of actin, tropomyosin and troponin in a 7 : 1 : 1 stoichiometry [39]. These studies showed that troponin is highly elongated and aligns with the actin and tropomyosin strands. In particular, the C-terminal region of TNI (residues 137–210), together with the troponin core, interacts with four of the seven actin subunits. The SUMOylation-mediated change at residue K177 may very well alter the interaction between myosin and actin as binding of myosin to actin is a multi-step process which is not only regulated both by calcium, but also by the hydrophobic properties of filament proteins.

Another possible explanation for the SUMOylation cascade-mediated changes in samples over-expressing SUMO-null TNI could be that SUMO modification of TnI has a role distinct from that involved in myofilament function, for example, as in Drosophila where SUMOylation regulates TnI’s ability to act in the nucleus as a factor to protect chromosome stability [35]. The literature contains other examples of SUMO-modifications which do not alter the recognised function of the substrate that include Aurora-B kinase where SUMO-modification does not influence kinase activity [40] and ataxin-3 where the modification does not alter the protein’s ability to aggregate [41]. SUMOylation has been shown to increase the stability of many proteins by reducing the likelihood of natural degradation by the proteasome [42], however we show that this is not the case for TnI (Figs 5 and 7). Sequestration of SUMO via conjugation to TnI K177 may prevent other more functionally relevant proteins in the vicinity of myofilament being SUMOylated and this block in SUMO availability may be reversed with over-expression of K177-R mutants allowing SUMOylation of other proteins that are germane in this context.

In conclusion, we present the first report of SUMOylation of TnI in human heart and suggest that the function of this modification may be an important but indirect one. Further investigation is required to better characterise the molecular mechanisms that influence cardiac physiology following over-expression of SUMO-null TNI.

## Materials and methods

### Human samples

Human heart samples (details outlined in Table 1) were obtained from the Gill Cardiovascular Biorepository at the University of Kentucky. Samples were procured from non-failing hearts from organ donors and from failing hearts when patients received a cardiac transplant. Patients and the families of the organ donors provided written consent. All procedures were approved by the local IRB. Details of the collection procedures have been published [43].

The group of samples from diseased hearts were somewhat heterogeneous, with patients diagnosed with ischaemic heart failure or chronic systolic heart failure. These samples were considered together as a single group for quantitative analysis.

### Porcine samples

Porcine samples were kindly gifted from Dr. Roger Hajjar (New York, USA). Pigs were subjected to left anterior descending artery balloon occlusion, an established model of MI [44].

### Animals

Both neonatal rat ventricular myocytes (NRVM) and adult rabbit ventricular myocytes (ARVM) were used. Sprague–Dawley rats and rabbits were handled in accordance with the UK Animals (Scientific Procedures) Act of 1986. All procedures were approved by the UK Home Office (PP7088535) and Glasgow University Ethics Review Committee. The animal research reported here adheres to the ARRIVE and Guide for the Care and Use of Laboratory Animals guidelines.

### Isolation and culture of rodent cardiac myocytes

Both neonatal rat ventricular myocytes (NRVM) and adult rabbit ventricular myocytes (ARVM) were used. Animals were handled in accordance with the UK Animals (Scientific Procedures) Act of 1986. NRVM were isolated as previously described [45]. Briefly, 1–4 days old Sprague–Dawley rats were sacrificed and the hearts were removed. Dissected ventricles were enzymatically digested and pre-plated to purify myocytes in suspension from adherent fibroblasts. Purified ventricular myocytes were cultured on gelatine-coated dishes in 4 : 1 Dulbecco’s modified Eagle’s medium (DMEM):Medium 199 (M199) supplemented with 10% horse serum (HS), 5% newborn calf serum (NCS), 1% l-glutamine and 1% penicillin/streptomycin. Following 24 h of culture, medium was aspirated and replaced with 4 : 1 DMEM: M199 supplemented with 5% HS, 0.5% NCS, 1% l-glutamine and 1% penicillin/streptomycin. ARVM were isolated as previously described from adult male New Zeal- and rabbits weighing between 3.0 and 4.0 kg [46]. Hearts were extracted and mounted on Langendorff perfusion rigs. Tissues were digested by retrograde perfusion of enzyme solution through the ascending aorta. Ventricles were excised and agitated to dissociate single cells. Ca^2+^ was gradually reintroduced to cells prior to culture on laminin-coated 35 mm glass bottom dishes (MatTek, Ashland, MA, USA) in M199 supplemented with 5 mM creatine, 5 mM carnitine, 5 mM taurine and 1% penicillin/streptomycin.

### DNA constructs, AAV vectors and cell transfection

Plasmids encoding TnI-UBC9 fusion proteins were created by inserting TnI into a UBC9 construct contained within a pcDNA3.1 vector. The TnI SUMO site mutation (KK177-8RR) was introduced via site directed mutagenesis (Agilent, Santa Clara, CA, USA) as per manufacturer’s instructions. Plasmid DNA was transfected into NRVM using Lipofectamine LTX (Thermo Fisher Scientific Inc., Waltham, MA, USA) as per manufacturer’s instructions. Viral vectors were used for the overexpression of TnI-HA proteins. Wildtype and mutant TnI-HA were inserted into E1/E3 deleted adenoviruses (AV) and purified (Welgen, Inc., Worcester, MA, USA). AVs were administered directly to cell culture medium.

### *In vitro* SUMOylation

*In vitro* SUMOylation assays (Enzo) were used as per manufacturer’s instructions. Briefly, all necessary components of the enzymatic SUMOylation cascade and cofactors were incubated with a putative target protein, which was within a cell lysate or immobilised on a peptide array. Resulting assays were then immunoblotted to determine whether SUMOylation had occurred.

### Immunoblotting

Western blots (WB) and peptide arrays (PA) were immunoblotted for various proteins. The following antibodies were used: GAPDH (1 : 5000 WB; Millipore, Burlington, MA, USA; MAB374), HA tag (1 : 500 IF, 1 : 1000 WB; Cell Signalling Technologies, Danvers, MA, USA; 2367), SUMO-TnI (1 : 100 PA, WB, custom antibody produced in rabbits against SUMOylated synthetic peptide N-HLKQVKKEDTEK-C; Badrilla, Leeds, UK), SUMO1 (1 : 1000 PA, WB, Enzo BML-PW9465-0025), TnI (1 : 500 IF, 1 : 1000 WB; Abcam, Waltham, MA, USA; ab27003), Donkey anti-mouse fluorescent secondary (1 : 5000 WB; Licor, Lincoln, NE, USA; 925–68 072), Donkey anti-rabbit fluorescent secondary (1 : 5000 WB; Licor 925–68 073), Goat anti-rabbit HRP secondary (1 : 5000 PA, WB; Sigma, Welwyn Garden City, UK; A6154), Alexa Fluor® 488 goat anti-mouse (1 : 500 IF; Invitrogen, Waltham, MA, USA; A21071), and Alexa Fluor® 647 goat anti-rabbit (1 : 500 IF; Invitrogen A21121).

### Peptide arrays

Immobilised 25-mer peptides were spotted onto cellulose membranes using an Autospot Robot ASS222 (Intavis®, Cologne, Germany) as previously described [47]. Arrays were subjected to *in vitro* SUMOylation Assay (Enzo) and subsequently immunoblotted using primary antibodies and HRP-conjugated secondary antibodies. Enhanced chemiluminescence (ECL) was used to visualise arrays and positive spots indicated antibody detection.

### Force measurements in skinned myocytes

Skinned ARVM were prepared using incubation with 0.5% Triton X-100 in relaxing solution (pH 7.0; in mmol L^−1^: free Mg^2+^ 1, KCl 145, EGTA 2, ATP 4, imidazole 10) and stored at −20 °C in 50% glycerol in relaxing solution prior to force measurements. Force measurements from single cells were recorded and analysed as previously described [26,48]. Briefly, isometric force measurements were recorded for 12 cells per condition from three separate cell isolations at a sarcomere length of 2.2 μm. The force-calcium relation of the cell was determined by measuring force development at varying calcium concentrations, ranging from pCa 9.0 to pCa4.5. Ca^2+^ sensitivity was defined as the calcium^+^ required to reach half-maximal force development (EC50). Cooperativity of the myofilaments was defined by the steepness of the sigmoidal force-pCa relationship (Hill slope). Passive tension of the myofilaments (*F*_passive_) was determined by a rapid 30% shortening of the cell at sarcomere length 2.2 μm.

### Proximity ligation assay

To visualise SUMOylation of TNI in endogenous systems, Duolink® proximity ligation assay (PLA) was employed using the manufacturer’s protocol and antibodies against cTNI and SUMO-TNI simultaneously.

### Contractility measurements

Contractility measurements were conducted using CellOPTIQ (Clyde Biosciences Ltd, Glasgow, UK) [25]. Cells were paced using electrodes at 40 V with 20 ms duration at a frequency of 1.2 Hz.

For each field, an 8-s recording at 100 frames per second was acquired.

using a 60× objective lens and Contractility Tool software. Between six and 10 fields of each monolayer were recorded over five experimental days using five separate NRVM isolations.

## Figures and Tables

**Table 1.** (A and B). Details of heart samples. Information about the heart samples utilised in this study including heart failure type (A) and primary diagnosis (B).

A			
Record ID	Case type	Heart failure type	Primary diagnosis
2B487 epi and endo	Organ donor		
3F6DC epi and endo	Heart transplant	Ischaemic	Ischaemic cardiomyopathy
4B3FA epi and endo	Organ donor		
4D931 epi and endo	Organ donor		
8E8D8 epi and endo	Heart transplant	Ischaemic	Ischaemic cardiomyopathy
14C39 Not specified	Heart transplant	Ischaemic	Ischaemic cardiomyopathy
97CDC Not specified	Heart transplant	Ischaemic	Ischaemic cardiomyopathy
632FD epi and endo	Organ donor		
5155D epi and endo	Organ donor		
8296A epi and endo	Heart transplant	Ischaemic	Ischaemic cardiomyopathy
31 331 epi and endo	Organ donor		
A7A62 Epi	Heart transplant	Ischaemic	Ischaemic heart failure
AF1FF epi and endo	Heart transplant	Ischaemic	Chronic systolic heart failure
B0644 epi and endo	Heart transplant	Ischaemic	Ischaemic cardiomyopathy
B8BE2 endo	Heart transplant	Ischaemic	Ischaemic heart failure
B23E3 epi and endo	Organ donor		
BC90C epi and endo	Organ donor		
C3B57 epi and endo	Heart transplant	Ischaemic	Ischaemic cardiomyopathy
CB8A5 epi and endo	Heart transplant	Ischaemic	Ischaemic cardiomyopathy
D0F54 epi and endo	Organ donor		
D612E epi and endo	Organ donor		
D8822 endo	Heart transplant	Ischaemic	Ischaemic heart failure and post-MI pericarditis
EF5CB Not specified		Ischaemic	Ischaemic heart failure
046E1 epi and endo	Heart transplant	Ischaemic	Ischaemic cardiomyopathy
05FF7 epi and endo	Heart transplant	Ischaemic	Ischaemic HFrEF
24 713 epi and endo	Organ donor		
5845F epi and endo	Heart transplant	Ischaemic	Ischaemic Cardiomyopathy s/p MI
6DB85 epi and endo	Heart transplant	Ischaemic	HFrEF Ischaemic cardiomyopathy
7 CE52 epi and endo	Heart transplant	Ischaemic	Ischaemic heart failure
8CB30 epi and endo	Organ donor		
9D7E9 epi and endo	Heart transplant	Ischaemic	Ischaemic cardiomyopathy
DA820 epi and endo	Heart transplant	Ischaemic	Ischaemic cardiomyopathy
F8EE8 epi and endo	Heart transplant	Ischaemic	Ischaemic cardiomyopathy
FC3CB epi and endo	Organ donor		
FE8E2 epi and endo	Heart transplant	Ischaemic	Chronic systolic HF

B		
Case type (primary diagnosis)	Patient numbers	Sample numbers
Health	13	26
Ischaemic cardiomyopathy	13	24
Ischaemic heart failure	4	5
Chromic systolic heart failure	1	2
Ischaemic heart failure and post-MI pericarditis	1	1
Ischaemic HFrEF	1	2
HFrEF from ischaemic cardiomyopathy	1	2
Chronic systolic HF	1	2
Total	35	64

## Data Availability

All raw data and materials will be made available following a reasonable request.

## Abbreviations

TNI: troponini I
TnT: troponin T
TnC: troponin C
SUMO: small ubiquitin-like modifier protein
SENP: deSUMOylating enzymes
HEK: human embryonic kidney
NRVM: neonatal rat ventricular myocytes
